# Antipsychotic medications and stroke in schizophrenia: A case-crossover study

**DOI:** 10.1371/journal.pone.0179424

**Published:** 2017-06-14

**Authors:** Wen-Yin Chen, Lian-Yu Chen, Hsing-Cheng Liu, Chi-Shin Wu, Shu-Yu Yang, Chun-Hung Pan, Shang-Ying Tsai, Chiao-Chicy Chen, Chian-Jue Kuo

**Affiliations:** 1 Taipei City Psychiatric Centre, Taipei City Hospital, Taipei, Taiwan; 2 Graduate Institute of Epidemiology and Preventive Medicine, National Taiwan University College of Public Health, Taipei, Taiwan; 3 Department of Psychiatry, National Cheng Kung University, Tainan, Taiwan; 4 Department of Psychiatry, School of Medicine, Taipei Medical University and Psychiatric Research Center, Taipei Medical University Hospital, Taipei, Taiwan; 5 Department of Psychiatry, National Taiwan University Hospital, Taipei, Taiwan; 6 Graduate Institute of Clinical Pharmacy, College of Pharmacy, Kaohsiung Medical University, Kaohsiung, Taiwan; 7 Department of Psychology, National Chengchi University, Taipei, Taiwan; 8 Department of Psychiatry, Mackay Memorial Hospital, Taipei, Taiwan and Department of Psychiatry, Mackay Medical College, Taipei, Taiwan; University of Texas Health Science Center at San Antonio Cancer Therapy and Research Center at Houston, UNITED STATES

## Abstract

**Background:**

The association between antipsychotic use and the risk of stroke in schizophrenic patients is controversial. We sought to study the association in a nationwide cohort with schizophrenia.

**Methods:**

Using a retrospective cohort of patients with schizophrenia (N = 31,976) derived from the Taiwan National Health Insurance Research Database, 802 new-onset cases of stroke were identified within 10 years of follow-up (from 2000 through 2010). We designed a case-crossover study using 14-day windows to explore the risk factors of stroke and the association between antipsychotic drugs and the risk of stroke. We analyzed the risks of individual antipsychotics on various subgroups of stroke including ischemic, hemorrhagic, and other strokes, and the risks based on the antipsychotic receptor-binding profile of each drug.

**Results:**

Use of any second-generation antipsychotic was associated with an increased risk of stroke (adjusted risk ratio = 1.45, *P* = .009) within 14 days while the use of any first-generation antipsychotic was not. Intriguingly, the use of any second-generation antipsychotic was associated with ischemic stroke but not hemorrhagic stroke. The antipsychotic receptor-binding profile analysis showed that the antihistamine 1 receptor was significantly associated with ischemic stroke (adjusted risk ratio = 1.72, *P* = .037), and the sensitivity analysis based on the 7-day window of exposure validated the association (adjusted risk ratio = 1.87, *P* = .015).

**Conclusions:**

Use of second-generation antipsychotic drugs appeared to be associated with an increased risk of ischemic stroke in the patients studied, possibly mediated by high affinity for histamine-1 receptor blockade. Further research regarding the underlying biological mechanism and drug safety is suggested.

## Introduction

Schizophrenia is a debilitating mental illness, and the lifespan of patients with schizophrenia is estimated to be 12–15 years shorter than that of the general population [1]. This reduction of lifespan could be due to comorbid physical illnesses such as stroke. A recent meta-analysis showed that schizophrenia patients appeared to be at greater risk of stroke [2]. Clinical comorbidity, such as diabetes, hypertension, and hyperlipidemia may be linked to the development of atherosclerosis, which is a pathogenesis shared with stroke [3]. Other factors that could contribute to the stroke event include unhealthy lifestyles, poor healthcare, and drug exposure [4, 5]. Antipsychotics are crucial for the treatment of schizophrenia, and drug safety is always of critical concern. The dose and duration of antipsychotic use for treating schizophrenia are even higher and longer than prescriptions for other psychiatric conditions. Therefore, the role of antipsychotic drugs in the causal relationship of stroke in patients with schizophrenia needs clarification.

Most studies on the effects of antipsychotics on stroke risk were conducted in elderly patients and were seldom specific to schizophrenia patients. The pooling of results of meta-analyses from several randomized, placebo-controlled clinical trials showed an increased risk of cerebrovascular events in patients with dementia receiving second-generation antipsychotics (SGAs) [6]. A self-controlled case-crossover study [5] demonstrated that both FGAs and SGAs were associated with the risk of stroke among elderly patients. In contrast, a population-based case-control study [7] showed that only the use of FGAs was slightly associated with the risk of stroke but not with the use of SGAs. Thus, the above studies showed inconsistent findings. Furthermore, we hypothesized that the previous findings in elderly populations should not be generalized directly to schizophrenia patients, which is a topic that still deserves further study.

In previous studies with case-control designs, the limitations have concerned unrecognized confounding factors, which could bias the estimation of the risks [8]. Prior studies [9, 10] have shown a temporal relationship between antipsychotic use and stroke as an acute effect (most significant within 7–14 days) instead of a long-term effect. Considering this knowledge gap, we applied a case-crossover design in our study such that the association of drug exposure and risk of stroke was identified an acute effect [9, 10]. The case-crossover design has been widely used [11, 12] when a brief exposure has caused a transient change in the risk of a rare acute-onset disease, in which a medication change was an exacerbating factor in acute stroke. The design only involved the cases, and intriguingly, self-matching of cases further eliminated the threat of control-selection bias and increased efficiency [13]. Therefore, this design has the advantage that the study subjects serve as their own controls and can remove unmeasured time-invariant confounders [11, 12].

In addition, the mechanism of antipsychotic-associated stroke remains speculative and may involve different pathophysiological pathways, ranging from the facilitation of thrombosis, pre-existing cardiovascular factors, sedation, and a common diathesis for strokes in psychiatric disorders[14]. However, there is little evidence to support these hypothetical mechanisms. The receptor-binding profiles of antipsychotics are associated with specific side effects. Therefore, these profiles can provide an opportunity to understand the mechanism of associations between receptor-binding of antipsychotics and stroke [8].

In this study, we assess the association between antipsychotic exposure and the risk of stroke by employing a large schizophrenia cohort derived from a nationwide population-based insurance claim dataset. The effects of antipsychotics on different types of stroke were evaluated, and a possible mechanism of action is hypothesized based on the receptor-binding profiles of the antipsychotics.

## Materials and methods

### Data sources

The National Health Institutes Research Database (NHIRD) in Taiwan is available to researchers upon application approval [15]. The NHIRD contains the Psychiatric Inpatient Medical Claims database (PIMC), which includes patients whose admitting diagnosis, based on the International Classification of Diseases, Ninth Revision (ICD-9), included codes ranging from 290 to 319 from January 1, 1996, to December 31, 2008 (*N* = 187,117). The veracity of the database is ensured by the periodic review and recertification of each hospital that provides psychiatric hospitalization in Taiwan. The Taiwan Joint Commission on Hospital Accreditation, an independent, non-governmental organization, oversees hospital accreditation. The accreditation for qualified psychiatric services requires board-certified psychiatrists to diagnose inpatients. In addition, this database has been the source for numerous epidemiological and clinical studies published in peer-reviewed journals [8, 10, 15].

For defining the longitudinal retrospective cohort with possible incident schizophrenia instead of prevalent schizophrenia, we first selected patients from the database with at least one psychiatric hospital admission between 2000 and 2008 but no psychiatric admissions between 1996 and 1999 to recruit the patients who had had their first incident of mental illness requiring psychiatric hospitalizations (*n* = 125,225). Then, the inclusion criteria for the subjects were at least one discharge diagnosis of schizophrenia (ICD-9-CM code 295.xx) with age at the first psychiatric admission ranging from 18 to 65 years (*n* = 33,024). All of the prescription records of these patients were retrieved from 2000 through 2010. Each prescription record contained the medications, dosage, and duration of the drug regimen. Patients who were diagnosed with stroke (ICD-9-CM codes 430.x to 436.x) before the first psychiatric admission (baseline) were excluded (*n* = 1048).

We enrolled patients with an incident stroke after their schizophrenia diagnosis as cases (n = 802), defined as those with a primary diagnosis of hospitalization for cerebrovascular events encoded as ICD-9-CM 430, 431, and 432 for hemorrhagic stroke, 433, 434, and 435 for ischemic stroke, and 436 for other types of stroke. The onset of stroke (index date) was defined as the date of the first-time hospitalization or using an emergent service, with a diagnosis of stroke. Fig A in S1 File presents the flowchart of subject enrollment. Sociodemographic and clinical characteristics were recorded, including age at first psychiatric admission, age at stroke event, Charlson comorbidity score [16, 17], the number of psychiatric admissions within one year before the index date and cumulative days of any antipsychotic use within one year before the index date.

### Ethics statement

The Institutional Review Board of Taipei City Hospital approved this case-crossover study using national claims data from the NHIRD. A waiver of informed consent was granted since the patient information was de-identified before the analysis. All researchers signed an agreement guaranteeing patient confidentiality before using the database. The National Health Research Institute in Taiwan established the NHIRD to provide data for research purposes. Please find the website (http://nhird.nhri.org.tw/en/) to request the information this study used, such as a point of contact and data files. The database constitutes medical claim files representative of the entire population in Taiwan.

### Case-crossover study

The case-crossover design is a standard method for assessing the effect of brief exposures on acute outcomes [13, 18], and thus, is suitable for our purpose to reveal the changes of antipsychotic exposures that vary over time and produce a transient change in the risk of an acute stroke within a short period. With this study design, each case subject served as their own control. We assessed potential factors during a 14-day window as in a similar previous study [10] and compared the 14-day case period (1–14 days just before the stroke) with four individual 14-day control periods (every 13 weeks: 92–105 days, 183–196 days, 274–287, and 365–378 days before the onset of stroke).

### Time-variant factors

According to the defined time windows, we assessed the association of stroke with the potential factors in schizophrenia. The potential factors comprised several physical illnesses including hypertension, non-hypertensive cardiovascular disease, diabetes mellitus, cancer, chronic hepatic disease, asthma, upper respiratory tract infection, and delirium. Additionally, concomitant medications included cardiovascular drugs, lipid-modifying agents, drugs used in diabetes, antithrombotic agents, corticosteroids, anti-Parkinson drugs, respiratory drugs, and benzodiazepines. The potential factors were selected as candidate risk factors due to their potential associations with the risk of stroke [19]. The identified risk factors also served as covariates for assessing the associations of antipsychotics, mood stabilizers, and antidepressants with the risk of stroke.

### Exposure to antipsychotics, mood stabilizers, and antidepressants

We retrieved prescription drug data on the use of antipsychotics, mood stabilizers, and antidepressants. We defined antipsychotics (N05A) according to the Anatomical Therapeutic Chemical Classification System (ATC/DDD Index 2015. http://www.whocc.no/atc_ddd_index/ [accessed June 6, 2015]) and classified them as either FGAs or SGAs.

We obtained the binding affinities for a variety of neurotransmitter receptors, including serotonin (5HT [hydroxytryptamine] 1A, 5HT2A, 5HT6, and 5HT7), dopamine (D2 and D4), histamine (H1), muscarinic (M1), and adrenergic receptors (A1 and A2) from the National Institute of Mental Health Psychoactive Drug Screening Program Ki Database [20]. Patients with antipsychotics for which the Ki value for binding affinity was not available in the database were categorized as others in the analyses of the neurotransmitter receptor-binding profiles and stroke risk. Frequently prescribed antipsychotics (more than 2.0% for each in the case period) including chlorpromazine, haloperidol, flupentixol, sulpiride, clozapine, olanzapine, quetiapine, zotepine, risperidone, amisulpride, and aripiprazole were assessed based on the antipsychotics’ dissociation constants for the affinities to a variety of receptors (Table A in S1 File). Each antipsychotic drug was categorized into either a high or a low group, based on the median values of the binding affinity for each neurotransmitter receptor.

### Statistical analysis

We conducted a two-step multivariate, conditional logistic regression analysis. First, we explored physical illnesses and concomitant medications as potential risk factors for stroke. We conducted the multivariate regression based on the strategy of backward variable selection. The variables with a modest association (*P* < .20) were retained in the final model. Second, we investigated the effects of individual antipsychotic drugs, mood stabilizers, and antidepressants on the risk of stroke. Because the prescription of psychiatric drugs in the different time windows could be confounding by indications with the presence of comorbid physical illnesses and concomitant medications[21], we conducted a propensity score adjusted regression analysis [22] to estimate the effect of the individual psychiatric drug on the risk of stroke.

Subgroup analyses were performed to estimate the effects of the antipsychotics on the various types of stroke by restricting the outcomes to ischemic, hemorrhagic, and other types of stroke due to their different underlying mechanisms. The receptor affinity of the antipsychotics on the risk of stroke was analyzed based on four categories (no use, high affinity, low affinity, and others), and we used the physical illnesses and concomitant drugs as covariates for the multiple regression analysis. All analyses were conducted using SAS software, version 9.2 (SAS Institute Inc., Cary, NC, USA), and a P-value of less than .05 was considered significant.

We further computed the odds ratios using two different time windows of 7 days and 28 days before the incident stroke for the sensitivity analysis.

## Results

### Characteristics of the study subjects

The study cohort included 31,976 schizophrenia patients, and 802 of them had incident strokes. The annual incidence of stroke was 34.6 cases per 10,000 person-years. Among the 802 cases, their age (SD) at first psychiatry admission was 50.0 (12.2) years and the mean (SD) time to the incident stroke was 3.9 (2.7) years during the 10-year follow-up period. Table 1 provides the demographic features and reveals that more than two-thirds of patients had the first stroke after their 46th year, and more than half (56.6%) of the patients with strokes were men. The types of stroke included ischemic (55.6%), hemorrhagic (29.3%), and others (15.1%). Most of the patients (93.7%) had Charlson comorbidity scores of less than three at their first psychiatry admission, and only 12.8% of patients showed no cumulative days of any antipsychotic use within one year before the incident stroke.

**Table 1:** Demographic and clinical characteristics of 802 patients with schizophrenia and first-time stroke, 2000–2010.

Characteristic	*N* (%)
Age at first admission (years)	
18–34	142 (17.7)
35–45	201 (25.1)
46–54	244 (30.4)
55–65	215 (26.8)
Age at first stroke (years)	
18–34	103 (12.8)
34–45	157 (19.6)
46–54	224 (27.9)
55–65	242 (30.2)
> 65	76 (9.5)
Sex	
Male	454 (56.6)
Female	348 (43.4)
Stroke type	
Ischemic	446 (55.6)
Hemorrhagic	235 (29.3)
Other	121 (15.1)
Charlson comorbidity index at first admission	
1	597 (74.4)
2	155 (19.3)
≥ 3	50 (6.2)
Number of psychiatric admissions within a year before index stroke (index date)	
0	577 (72.0)
1	164 (20.4)
2	44 (5.5)
≥ 3	17 (2.1)
Cumulative days of any antipsychotic use within 1 year before the incident stroke (index date)	
0	103 (12.8)
1–7	9 (1.1)
8–28	24 (3.0)
29–56	31 (3.9)
56–150	122 (15.2)
150–365	513 (64.0)

### Factors associated with incident stroke

In the multivariate conditional logistic regression analyses (Table 2), several factors exhibited a significantly higher risk of stroke, including cardiovascular disease (except hypertension) (adjusted RR = 2.32, *P* < .001) and diabetes mellitus (RR = 1.54, *P* = .005). Concomitant medications including systemic antihistamines (RR = 1.70, *P* < .001) and benzodiazepines (RR = 1.44, *P* = .004) also increased the risk of stroke.

**Table 2:** Association between the risk of stroke and physical illnesses and concomitant drugs within the 14-day case period in schizophrenia patients with first-time stroke.

Characteristic	Case period *N* (%)	Control period 1 *N* (%)	Control period 2 *N* (%)	Control period 3 *N* (%)	Control period 4 *N* (%)	Crude risk ratio^a^	*P*-value^a^	Adjusted risk ratio^b^	*P*-value^b^
All patients (*n* = 802)									
Physical illnesses									
Cardiovascular disease (except H/T)	122 (15.2)	89 (11.1)	71 (8.9)	65 (8.1)	71 (8.9)	2.90**	< .001	2.32**	< .001
Hypertension	120 (15.0)	113 (14.1)	91 (11.4)	101 (12.6)	100 (12.5)	1.61*	.007		
Diabetes mellitus	116 (14.5)	112 (14.0)	90 (11.2)	92 (11.5)	85 (10.6)	2.33**	< .001	2.23**	.005
Cancer	13 (1.6)	13 (1.6)	11 (1.4)	10 (1.2)	6 (0.7)	2.91	.120		
Chronic hepatic disease	39 (4.9)	33 (4.1)	30 (3.7)	25 (3.1)	27 (3.4)	1.80*	.025	1.44	.183
Asthma	12 (1.5)	9 (1.1)	13 (1.6)	9 (1.1)	14 (1.7)	1.16	.764		
Upper respiratory tract infection	95 (11.9)	81 (10.1)	81 (10.1)	77 (9.6)	85 (10.6)	1.62*	.001		
Delirium	0 (0.0)	1 (0.1)	1 (0.1)	1 (0.1)	1 (0.1)	0.00	.972		
Concomitant drugs									
Cardiovascular drugs									
Antihypertensive drugs	29 (3.6)	27 (3.4)	21 (2.6)	21 (2.6)	19 (2.4)	1.89	.054		
Beta-blocking agents	209 (26.1)	190 (23.7)	175 (21.8)	163 (20.3)	157 (19.6)	1.83**	< .001	1.30	.074
Calcium channel blockers	115 (14.3)	102 (12.7)	84 (10.5)	79 (9.9)	86 (10.7)	1.95**	< .001	1.36	.091
Agents acting on the rennin-angiotensin system	82 (10.2)	68 (8.5)	64 (8.0)	61 (7.6)	58 (7.2)	1.93*	.001		
Lipid-modifying agents	27 (3.4)	24 (3.0)	26 (3.2)	24 (3.0)	26 (3.2)	1.17	.613		
Drugs used in diabetes	104 (13.0)	104 (13.0)	94 (11.7)	94 (11.7)	88 (11.0)	1.50	.090	0.66	.168
Antithrombotic agents	74 (9.2)	59 (7.4)	50 (6.2)	39 (4.9)	46 (5.7)	2.47**	< .001		
Corticosteroids for systemic use	35 (4.4)	37 (4.6)	31 (3.9)	21 (2.6)	18 (2.2)	1.46	.104		
Nasal preparations	48 (6.0)	30 (3.7)	35 (4.4)	32 (4.0)	30 (3.7)	1.77**	.005	1.46	.072
Drugs for obstructive airway diseases	60 (7.5)	50 (6.2)	49 (6.1)	48 (6.0)	42 (5.2)	1.47*	.042		
Cough and cold preparations	144 (18.0)	129 (16.1)	130 (16.2)	113 (14.1)	116 (14.5)	1.34*	.022		
Antihistamines for systemic use	149 (18.6)	108 (13.5)	102 (12.7)	91 (11.4)	87 (10.9)	2.00**	< .001	1.70**	< .001
Anti-Parkinson drugs	409 (51.0)	403 (50.3)	404 (50.4)	360 (44.9)	377 (47.0)	1.32*	.024		
Respiratory drugs	230 (28.7)	188 (23.4)	180 (22.4)	168 (21.0)	165 (20.6)	1.72**	< .001		
Benzodiazepines	500 (62.3)	478 (59.6)	461 (57.5)	436 (54.4)	439 (54.7)	1.72**	< .001	1.44**	.004

*p < .05,

**p < .01.

H/T = hypertension.

^a^Estimated using univariate conditional logistic regression.

^b^Estimated using multivariate conditional logistic regression. Adjusted for physical illnesses and concomitant medications that remained in the final model.

### Effect of antipsychotics, mood stabilizers, and antidepressants on the risk of stroke

Based on the propensity score adjusted analysis, Table 3 reveals that FGAs are not associated with the risk of stroke while SGAs are significantly associated with an elevated risk of stroke (adjusted RR = 1.45, *P* = .009). Among the individual SGAs, quetiapine and zotepine were significantly associated with the risk of stroke. Among the mood stabilizers, valproic acid was the only one with an increased likelihood of stroke (adjusted RR = 2.68, *P* < .001). Use of antidepressants was not significantly associated with the risk of stroke. Regarding possible differences due to sex, further analyses show equivalent effect sizes of the adjusted risk ratios for the drugs studied in both men and women. Although some analyses showed adjusted risk ratios in men were significant, they were insignificant in women. For example, the adjusted risk ratios of any SGA on the stroke risk in men was 1.48 (p<0.05) and that of women was 1.43 (p = 0.089).

**Table 3:** Association between the risk of stroke and the use of antipsychotic drugs within the 14-day case period in schizophrenia patients with first-time stroke.

Characteristic	Case period *N* (%)	Control period 1 *N* (%)	Control period 2 *N* (%)	Control period 3 *N* (%)	Control period 4 *N* (%)	Adjusted risk ratio^a^	*P*-value^a^
All patients (*n* = 802)							
Any first-generation antipsychotic	304 (37.9)	289 (36.0)	289 (36.0)	272 (33.9)	275 (34.3)	1.04	.800
Chlorpromazine	31 (3.9)	29 (3.6)	37 (4.6)	25 (3.1)	27 (3.4)	0.86	.605
Haloperidol	124 (15.5)	112 (14.0)	109 (13.6)	101 (12.6)	95 (11.9)	1.13	.467
Flupentixol	38 (4.7)	35 (4.4)	37 (4.6)	29 (3.6)	30 (3.7)	1.10	.750
Sulpiride	115 (14.3)	116 (14.5)	106 (13.2)	112 (14.0)	112 (14.0)	0.89	.520
Any second-generation antipsychotic	345 (43.0)	319 (39.8)	308 (38.4)	285 (35.5)	293 (36.5)	1.45**	.009
Clozapine	48 (6.0)	49 (6.1)	48 (6.0)	39 (4.9)	39 (4.9)	1.24	.552
Olanzapine	52 (6.5)	47 (5.9)	46 (5.7)	44 (5.5)	48 (6.0)	1.16	.587
Quetiapine	57 (7.1)	49 (6.1)	51 (6.4)	42 (5.2)	39 (4.9)	1.63*	.049
Zotepine	43 (5.4)	35 (4.4)	30 (3.7)	31 (3.9)	34 (4.2)	1.78*	.045
Risperidone	137 (17.1)	118 (14.7)	121 (15.1)	105 (13.1)	124 (15.5)	1.21	.243
Amisulpride	24 (3.0)	25 (3.1)	26 (3.2)	19 (2.4)	22 (2.7)	0.97	.929
Aripiprazole	16 (2.0)	9 (1.1)	10 (1.3)	12 (1.5)	11 (1.4)	2.12	.093
Mood stabilizer	127 (15.8)	106 (13.2)	104 (13.0)	96 (12.0)	99 (12.3)	2.05**	< .001
Lithium	19 (2.4)	18 (2.2)	16 (2.0)	15 (1.9)	17 (2.1)	1.57	.406
Valproic acid	83 (10.4)	67 (8.4)	64 (8.0)	60 (7.5)	55 (6.9)	2.68**	< .001
Carbamazepine	30 (3.7)	25 (3.1)	27 (3.4)	24 (3.0)	30 (3.7)	1.24	.557
Antidepressant	133 (16.6)	124 (15.5)	112 (14.0)	110 (13.7)	108 (13.5)	1.40	.064

*p < .05,

**p < .01

^a^Estimated using propensity score adjusted analysis. Adjusted for physical illnesses and concomitant medications that remained in the final model in Table 2.

### Subgroup analysis and effect of antipsychotic receptor-affinity profiles

**Fig 1:**
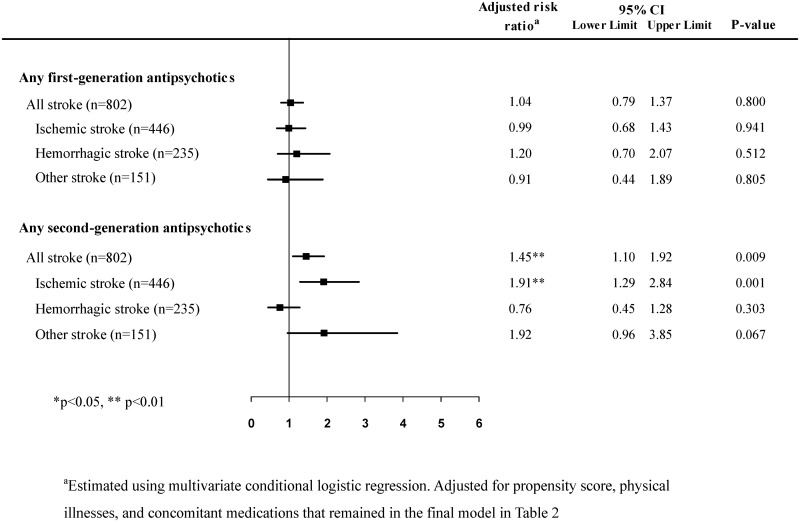
Association between the risk of stroke and the use of antipsychotic drugs within the 14-day case period in schizophrenia patients with first-time stroke.

The subgroup analyses revealed SGAs were significantly associated with ischemic stroke but not hemorrhagic stroke or other types of strokes (Fig 1). We further analyzed the risk of ischemic stroke based on the antipsychotic drug receptor-binding affinities in Table 4. After adjusting for the covariates, only a high affinity for histamine-1 receptor was associated with increased ischemic stroke risk. There were no significant associations for hemorrhagic and other types of strokes (Tables B and C in S1 File).

**Table 4:** Risk of ischemic stroke with antipsychotic use within the 14-day risk period according to the receptor-binding profile of the antipsychotics (*n* = 446).

Receptor-binding profile	Case period *N* (%)	Control period 1 *N* (%)	Control period 2 *N* (%)	Control period 3 *N* (%)	Control period 4 *N* (%)	Adjusted risk ratio^a^	*P*-value^a^
5HT1A							
No use	125 (28.0)	145 (32.5)	155 (34.8)	169 (37.9)	151 (33.9)	-	-
Other	8 (1.8)	13 (2.9)	10 (2.2)	12 (2.7)	13 (2.9)	0.52	.324
Low	136 (30.5)	130 (29.2)	129 (28.9)	123 (27.6)	129 (28.9)	1.24	.380
High	177 (39.7)	158 (35.4)	152 (34.1)	142 (31.8)	153 (34.3)	1.52	.090
5HT2A							
No use	125 (28.0)	145 (32.5)	155 (34.8)	169 (37.9)	151 (33.9)	-	-
Other	8 (1.8)	13 (2.9)	10 (2.2)	12 (2.7)	13 (2.9)	0.50	.298
Low	187 (41.9)	173 (38.8)	166 (37.2)	156 (35.0)	156 (35.0)	1.41	.154
High	126 (28.3)	115 (25.8)	115 (25.8)	109 (24.4)	126 (28.3)	1.34	.246
5HT6							
No use	125 (28.0)	145 (32.5)	155 (34.8)	169 (37.9)	151 (33.9)	-	-
Other	8 (1.8)	13 (2.9)	10 (2.2)	12 (2.7)	13 (2.9)	0.48	.267
Low	246 (55.2)	221 (49.6)	211 (47.3)	200 (44.8)	213 (47.8)	1.44	.104
High	67 (15.0)	67 (15.0)	70 (15.7)	65 (14.6)	69 (15.5)	1.10	.749
5HT7							
No use	125 (28.0)	145 (32.5)	155 (34.8)	169 (37.9)	151 (33.9)	-	-
Other	8 (1.8)	13 (2.9)	10 (2.2)	12 (2.7)	13 (2.9)	0.50	.299
Low	212 (47.5)	192 (43.1)	189 (42.4)	180 (40.4)	182 (40.8)	1.38	.163
High	101 (22.7)	96 (21.5)	92 (20.6)	85 (19.1)	100 (22.4)	1.36	.249
D2							
No use	125 (28.0)	145 (32.5)	155 (34.8)	169 (37.9)	151 (33.9)	-	-
Other	8 (1.8)	13 (2.9)	10 (2.2)	12 (2.7)	13 (2.9)	0.51	.308
Low	186 (41.7)	173 (38.8)	160 (35.9)	159 (35.7)	159 (35.7)	1.54	.077
High	127 (28.5)	115 (25.8)	121 (27.1)	106 (23.8)	123 (27.6)	1.21	.458
D4							
No use	125 (28.0)	145 (32.5)	155 (34.8)	169 (37.9)	151 (33.9)	-	-
Other	8 (1.8)	13 (2.9)	10 (2.2)	12 (2.7)	13 (2.9)	0.50	.301
Low	95 (21.3)	96 (21.5)	88 (19.7)	90 (20.2)	90 (20.2)	1.32	.308
High	218 (48.9)	192 (43.1)	193 (43.3)	175 (39.2)	192 (43.1)	1.40	.146
H1							
No use	125 (28.0)	145 (32.5)	155 (34.8)	169 (37.9)	151 (33.9)	-	-
Other	8 (1.8)	13 (2.9)	10 (2.2)	12 (2.7)	13 (2.9)	0.54	.353
Low	152 (34.1)	143 (32.1)	142 (31.8)	136 (30.5)	153 (34.3)	1.20	.444
High	161 (36.1)	145 (32.5)	139 (31.2)	129 (28.9)	129 (28.9)	1.72*	.037
M1							
No use	125 (28.0)	145 (32.5)	155 (34.8)	169 (37.9)	151 (33.9)	-	-
Other	8 (1.8)	13 (2.9)	10 (2.2)	12 (2.7)	13 (2.9)	0.49	.286
Low	14 (3.1)	12 (2.7)	12 (2.7)	9 (2.0)	11 (2.5)	2.19	.129
High	299 (67.0)	276 (61.9)	269 (60.3)	256 (57.4)	271 (60.8)	1.35	.180
A1							
No use	125 (28.0)	145 (32.5)	155 (34.8)	169 (37.9)	151 (33.9)	-	-
Other	8 (1.8)	13 (2.9)	10 (2.2)	12 (2.7)	13 (2.9)	0.52	.332
Low	135 (30.3)	134 (30.0)	127 (28.5)	126 (28.3)	131 (29.4)	1.23	.406
High	178 (39.9)	154 (34.5)	154 (34.5)	139 (31.2)	151 (33.9)	1.55	.073
A2							
No use	125 (28.0)	145 (32.5)	155 (34.8)	169 (37.9)	151 (33.9)	-	-
Other	8 (1.8)	13 (2.9)	10 (2.2)	12 (2.7)	13 (2.9)	0.50	.303
Low	187 (41.9)	175 (39.2)	176 (39.5)	163 (36.6)	169 (37.9)	1.24	.371
High	126 (28.3)	113 (25.3)	105 (23.5)	102 (22.9)	113 (25.3)	1.63	.060

Receptors: A1, adrenergic alpha 1; A2, adrenergic alpha 2; D2, dopamine 2; D4, dopamine 4; H1, histamine 1; 5HT1A, serotonin 1A; 5HT2A, serotonin 2A; 5HT6, serotonin 6; 5HT7, serotonin 7; M1, muscarinic 1.

*p< .05

^a^Estimated using multivariate conditional logistic regression. Adjusted for physical illnesses and concomitant medications that remained in the final model in Table 2.

The results for the 7- and 14-day time windows as the case and control periods are grossly consistent in the sensitivity analysis, which indicate the robustness of our findings (Table D in S1 File). Compared with patients who did not use antipsychotics, the adjusted RRs for ischemic stroke for the 7-day and 14-day windows for high-affinity histamine-1 receptor were 1.87 (*P* = .015) and 1.72 (*P* = .037), respectively. The results using the 28-day time window as the case and control periods showed no significant association with any receptors (Table E in S1 File). The findings imply an acute or subacute effect (limited to 14 days) for antipsychotic drug use on the risk of ischemic stroke.

## Discussion

### Main findings

In our study, the annual incidence of stroke in the patients with schizophrenia aged 18–65 years was 34.6 cases per 10,000 person-years. According to a prior study in Taiwan [23], the annual incidence of first-ever stroke is 21.3 per 10,000 person-years for people aged 36–64 years in the general population. Evidence indicates that the incidence of stroke increases with age, therefore, after considering the age stratification, our findings are consistent with the previously published literature [2] and reveal an increased risk of stroke in schizophrenia patients compared with the general population.

Our study is the first to apply a case-crossover design in the study of schizophrenia patients to investigate the association between antipsychotics and the risk of stroke. We found that any acute use (within two weeks before the onset of a stroke) of SGAs was associated with a 1.45-fold increased risk of stroke after adjusting for confounding factors. Higher stroke risk was observed in schizophrenia patients treated with quetiapine, zotepine, or valproic acid. Intriguingly, any use of an SGA was associated with ischemic stroke but not hemorrhagic stroke. Antipsychotics with higher binding affinity to H1 receptors could be the mechanism associated with the risk of ischemic stroke. Additionally, our results revealed that exposure to antihistamines and benzodiazepines increased the risk of stroke in schizophrenic patients.

### Drug exposure and the risk of stroke

We found no previous research directly linking the systemic use of antihistamines to the likelihood of stroke in human beings. An animal study [24] revealed that histamine interacted simultaneously with histamine-1 receptors on the endothelium and histamine-2 receptors on vascular muscle to mediate relaxation of the vessel. An in vitro study revealed a modulating interaction for histamine and second messengers in endothelium-dependent responses of microvascular compartments in the human brain [25]. Therefore, the systemic use of an antihistamine could interfere with histamine-mediated vasoconstriction or relaxation, which could lead to an increased risk of stroke. The identified association between antihistamine and risk of stroke could be related to one of the significant findings of this study, which showed higher antipsychotic drug receptor-binding affinity to histamine-1 receptors was associated with the risk of ischemic stroke.

Our results also suggest a possible association between benzodiazepine use and the risk of stroke. Several explanations for this association are that brain blood flow and glucose consumption decrease following administration of a GABA agonist [26], and sedation with benzodiazepines transiently allows the re-emergence of focal motor deficits in patients with prior stroke [27]. In addition, our findings show that valproic acid significantly increases the risk of stroke in schizophrenia patients. Valproic acid can affect both pro-coagulatory and anticoagulatory factors [28]. Time-dependent thickening of the carotid artery intima-media was observed in epileptic adults treated with valproic acid monotherapy, which could contribute to the acceleration of atherosclerosis [29], leading to stroke. Since valproic acid is widely used in patients with bipolar disorder [30], the risk deserves future studies in bipolar populations.

Among the individual SGAs, we confirmed that quetiapine and zotepine were associated with an increased risk of stroke. Quetiapine can cause weight gain as it blocks histamine-1 receptors, which also contributes to sedation, especially when combined with its M1-antimuscarinic and alpha-1 adrenergic antagonist properties [31]. Histamine 1 and alpha-1 adrenergic antagonist properties are also prominent in zotepine [31]. Sedation-related adverse effects may be the mediating factors of the association.

### Receptor-binding profile of antipsychotics and risk of ischemic stroke

Most previous studies have pooled outcomes of various types of stroke together. However, ischemic stroke and hemorrhagic stroke have distinctly different mechanisms [32]. Subgroup analysis in this study revealed that any use of an SGA was significantly associated with ischemic stroke but not hemorrhagic stroke or other types of stroke. We further conducted an analysis to identify a biological basis for the association between antipsychotics and the risk of ischemic stroke based on drug receptor-binding profiles.

We demonstrated that antipsychotics inducing histamine type 1 blockade were associated with a transient increase in the risk of ischemic stroke. This finding is reasonable in the case-crossover design, which is appropriate for short-term exposures of effect. The risk decreased from the 7-day time window to the 14-day window and diminished by the 28-day window, which implies that the mechanism of antihistamine action on ischemic stroke is an immediate or subacute, rather than a long-term process. Somnolence appeared to be a common side effect of antipsychotics with a high affinity to histamine-1 receptor blockade [31]. Excessive sedation could lead to venous stasis or dehydration and hemoconcentration, which might predispose a patient to ischemic stroke. Both quetiapine and zotepine are high-affinity histamine 1–receptor blockers. These findings corroborate our discussion about systemic antihistamine use increasing stroke risk in schizophrenic patients. Further physiological studies are needed to confirm these suggested mechanisms.

### Limitations

Several limitations should be considered in our study. First, despite most stroke patients undergoing image testing for diagnosis, the actual reports of the imaging examinations were unavailable in the NHIRD [8]. Therefore, we only categorized stroke into ischemic, hemorrhagic, and other types; we were unable to explore the associations between antipsychotic use and specific stroke subtypes [32]. Second, the receptor binding affinities of the antipsychotics were measured in in vitro studies; therefore, the actual activity of the antipsychotics on the receptors in the human brain deserves further clarification. Third, due to the use of claims databases in this study, there was no information regarding the potential confounding factors such as obesity, smoking, other unhealthy lifestyles or drug compliance. However, we used a self-controlled study design to adjust for such unmeasured variables.

### Conclusions

Higher stroke risk was observed in schizophrenia patients acutely treated with quetiapine, zotepine, and valproic acid. Furthermore, use of any SGA was associated with an acutely increased risk of ischemic strokes but not hemorrhagic strokes in patients with schizophrenia. Mediation by high affinity for histamine-1 receptor blockade of antipsychotics is a possible mechanism. Further research is needed regarding the underlying biological mechanisms and drug safety.

## Supporting information

S1 File **Fig A**. Study flow diagram. **Table A**. Receptor binding affinity (Pki) for antipsychotic drugs. **Table B**. Risk of hemorrhagic stroke with antipsychotic use within 14-day risk period by the receptor-binding profiles of the antipsychotic drugs (n = 235). **Table C**. Risk of other stroke with antipsychotic use within 14-day risk period by the receptor-binding profiles of the antipsychotic drugs (n = 121). **Table D**. Risk of ischemic stroke with antipsychotic use within 7-Day risk period by the receptor-binding profiles of the antipsychotic drugs (n = 446). **Table E**. Risk of ischemic stroke with antipsychotic use within 28-day risk period by the receptor-binding profiles of antipsychotic drugs (n = 446).
(DOC)
