# Correction: Antipsychotic medications and stroke in schizophrenia: A case-crossover study

**DOI:** 10.1371/journal.pone.0217323

**Published:** 2019-05-21

**Authors:** Wen-Yin Chen, Lian-Yu Chen, Hsing-Cheng Liu, Chi-Shin Wu, Shu-Yu Yang, Chun-Hung Pan, Shang-Ying Tsai, Chiao-Chicy Chen, Chian-Jue Kuo

There are errors in the author affiliations. The correct affiliations are as follows:

Wen-Yin Chen^1,2^, Lian-Yu Chen^1,3^, Hsing-Cheng Liu^1,4,5^, Chi-Shin Wu^6^, Shu-Yu Yang^1,7^, Chun-Hung Pan^1,8^, Shang-Ying Tsai^4,5^, Chiao-Chicy Chen^4,5,9^, Chian-Jue Kuo^1,4,5^

**1** Taipei City Psychiatric Centre, Taipei City Hospital, Taipei, Taiwan, **2** Graduate Institute of Epidemiology and Preventive Medicine, National Taiwan University College of Public Health, Taipei, Taiwan, **3** Department of Psychiatry, National Cheng Kung University, Tainan, Taiwan, **4** Department of Psychiatry, School of Medicine, College of Medicine, Taipei Medical University, **5** Psychiatric Research Center, Taipei Medical University Hospital, Taipei, Taiwan, **6** Department of Psychiatry, National Taiwan University Hospital, Taipei, Taiwan, **7** Graduate Institute of Clinical Pharmacy, College of Pharmacy, Kaohsiung Medical University, Kaohsiung, Taiwan, **8** Department of Psychology, National Chengchi University, Taipei, Taiwan, **9** Department of Psychiatry, Mackay Memorial Hospital, Taipei, Taiwan and Department of Psychiatry, Mackay Medical College, Taipei, Taiwan.
