# Supplementary material for: Antipsychotic medications and stroke in schizophrenia: A case-crossover study
Source: PLoS One. 2017 Jun 14;12(6):e0179424. doi: 10.1371/journal.pone.0179424 (PMC5470719; doi:10.1371/journal.pone.0179424)
Supplement: S1 File — Fig A. Study flow diagram. Table A. Receptor binding affinity (Pki) for antipsychotic drugs. Table B. Risk of hemorrhagic stroke with antipsychotic use within 14-day risk period by the receptor-binding profiles of the antipsychotic drugs (n = 235). Table C. Risk of other stroke with antipsychotic use within 14-day risk period by the receptor-binding profiles of the antipsychotic drugs (n = 121). Table D. Risk of ischemic stroke with antipsychotic use within 7-Day risk period by the receptor-binding profiles of the antipsychotic drugs (n = 446). Table E. Risk of ischemic stroke with antipsychotic use within 28-day risk period by the receptor-binding profiles of antipsychotic drugs (n = 446). (DOC) [file pone.0179424.s001.doc]

S1 Fig A. Study flow diagram.

Patients with the consistent diagnosis of schizophrenia (ICD-9 code: 295.**) (*n* = 38,120)

Patients between 18 and 65 years of age in the final study cohort (*n* = 33,024)

Final case subjects: patients with stroke incident hospitalized or emergent stroke (*n* = 802) after the first admission for schizophrenia

Broad search through all claims data from 2000 to 2010 for the longitudinal follow-up

Nationwide Psychiatric Inpatient Medical Claims (PMID) in Taiwan (ICD-9 code of 290.**–319.**) (1996–2008) (*N* = 187,117)

Excluding patients with at least one diagnosis of mood disorder (ICD-9 code: 296.**) from 1996-1999 (*n* = 87,105)

Patients first discharged with mental disorder diagnosis (ICD-9 code of 290.**–319.**), from 2000–2008 (*N* = 125,225) (no psychiatric admissions between 1996 and 1999)

Excluding the inpatient, outpatient, and emergency patients that had a stroke before the first admission date for schizophrenia (*n* = 1,048). The final study cohort (*n* = 31,976).

Hemorrhagic stroke (*n* = 235)

Ischaemic stroke (*n* = 446)

Other stroke (*n* = 121)

S1 Table A. Receptor binding affinity (Pki) for antipsychotic drugs

| Antipsychotic class and agent | 5HT1A | 5HT2A | 5HT6 | 5HT7 | D2 | D4 | H1 | M1 | A1 | A2 |
| --- | --- | --- | --- | --- | --- | --- | --- | --- | --- | --- |
| First-generation antipsychotics |  |  |  |  |  |  |  |  |  |  |
| Chlorpromazine | 5.67 | 7.96 | 7.77 | 7.55 | 8.28 | 7.94 | 8.51 | 7.49 | 8.59 | 6.12 |
| Haloperidol | 5.68 | 6.81 | 5.29 | 6.42 | 8.92 | 8.26 | 5.77 | 5.00 | 7.77 | 6.22 |
| Flupentixol | 5.10 | 7.06 | - | - | 8.82 | 6.88 | 9.07 | - | - | - |
| Sulpiride | 5.00 | 5.00 | 5.30 | 5.30 | 7.84 | 6.46 | 5.00 | 5.00 | 5.00 | 5.10 |
|  |  |  |  |  |  |  |  |  |  |  |
| Second-generation antipsychotics |  |  |  |  |  |  |  |  |  |  |
| Clozapine | 6.91 | 7.80 | 7.87 | 7.75 | 7.27 | 7.62 | 8.95 | 7.79 | 8.17 | 6.80 |
| Olanzapine | 5.64 | 8.62 | 8.09 | 6.98 | 7.28 | 7.73 | 8.66 | 7.59 | 7.36 | 6.55 |
| Quetiapine | 6.40 | 6.04 | 6.02 | 6.51 | 6.39 | 5.69 | 8.16 | 6.87 | 8.09 | 7.10 |
| Zotepine | 6.33 | 8.57 | 8.22 | 7.92 | 7.60 | 7.74 | 8.49 | 5.00 | 8.14 | 6.74 |
| Risperidone | 6.37 | 9.23 | 5.69 | 8.37 | 8.24 | 8.13 | 7.70 | 5.00 | 8.57 | 8.10 |
| Amisulpride | 5.00 | 5.08 | 5.38 | 7.94 | 8.89 | 5.63 | 5.00 | 5.00 | 5.00 | 5.80 |
| Aripiprazole | 8.25 | 8.06 | 6.24 | 8.00 | 9.02 | 6.29 | 7.54 | 5.17 | 7.59 | 7.13 |
|  |  |  |  |  |  |  |  |  |  |  |

Receptors: A1, adrenergic alpha 1; A2, adrenergic alpha 2; D2, dopamine 2; D4, dopamine 4; H1, histamine 1; 5HT1A, serotonin 1A; 5HT2A, serotonin 2A; 5HT6, serotonin 6; 5HT7, serotonin 7; M1, muscarinic 1.

^a^A minimal (pKi) value of 5.0 was used for low binding affinity.

S1 Table B. Risk of hemorrhagic stroke with antipsychotic use within 14-day risk period by the receptor-binding profiles of the antipsychotic drugs (n = 235)

| Receptor-binding profile | Case  period  *N* (%) | Control period 1  *N* (%) | Control period 2  *N* (%) | Control  period 3  *N* (%) | Control period 4  *N* (%) | Adjusted risk ratio^a^ | *P* value^a^ |
| --- | --- | --- | --- | --- | --- | --- | --- |
| 5HT1A |  |  |  |  |  |  |  |
| No use | 96 (40.9) | 88 (37.5) | 91 (38.7) | 98 (41.7) | 110 (46.8) | - | - |
| Other | 7 (3.0) | 10 (4.3) | 6 (2.6) | 8 (3.4) | 9 (3.8) | 0.49 | .329 |
| Low | 70 (29.8) | 66 (28.1) | 64 (27.2) | 56 (23.8) | 52 (22.1) | 1.14 | .683 |
| High | 62 (26.4) | 71 (30.2) | 74 (31.5) | 73 (31.1) | 64 (27.2) | 0.65 | .200 |
| 5HT2A |  |  |  |  |  |  |  |
| No use | 96 (40.9) | 88 (37.5) | 91 (38.7) | 98 (41.7) | 110 (46.8) | - | - |
| Other | 7 (3.0) | 10 (4.3) | 6 (2.6) | 8 (3.4) | 9 (3.8) | 0.50 | .334 |
| Low | 81 (34.5) | 81 (34.5) | 84 (35.7) | 78 (33.2) | 70 (29.8) | 0.88 | .689 |
| High | 51 (21.7) | 56 (23.8) | 54 (23.0) | 51 (21.7) | 46 (19.6) | 0.85 | .634 |
| 5HT6 |  |  |  |  |  |  |  |
| No use | 96 (40.9) | 88 (37.5) | 91 (38.7) | 98 (41.7) | 110 (46.8) | - | - |
| Other | 7 (3.0) | 10 (4.3) | 6 (2.6) | 8 (3.4) | 9 (3.8) | 0.50 | .340 |
| Low | 109 (46.4) | 111 (47.2) | 115 (48.9) | 106 (45.1) | 99 (42.1) | 0.83 | .534 |
| High | 23 (9.8) | 26 (11.1) | 23 (9.8) | 23 (9.8) | 17 (7.2) | 1.07 | .878 |
| 5HT7 |  |  |  |  |  |  |  |
| No use | 96 (40.9) | 88 (37.5) | 91 (38.7) | 98 (41.7) | 110 (46.8) | - | - |
| Other | 7 (3.0) | 10 (4.3) | 6 (2.6) | 8 (3.4) | 9 (3.8) | 0.52 | .358 |
| Low | 86 (36.6) | 86 (36.6) | 89 (37.9) | 82 (34.9) | 71 (30.2) | 0.95 | .877 |
| High | 46 (19.6) | 51 (21.7) | 49 (20.9) | 47 (20.0) | 45 (19.2) | 0.74 | .409 |
| D2 |  |  |  |  |  |  |  |
| No use | 96 (40.9) | 88 (37.5) | 91 (38.7) | 98 (41.7) | 110 (46.8) | - | - |
| Other | 7 (3.0) | 10 (4.3) | 6 (2.6) | 8 (3.4) | 9 (3.8) | 0.51 | .345 |
| Low | 71 (30.2) | 71 (30.2) | 62 (26.4) | 64 (27.2) | 61 (26.0) | 1.04 | .896 |
| High | 61 (26.0) | 66 (28.1) | 76 (32.3) | 65 (27.7) | 55 (23.4) | 0.71 | .309 |
| D4 |  |  |  |  |  |  |  |
| No use | 96 (40.9) | 88 (37.5) | 91 (38.7) | 98 (41.7) | 110 (46.8) | - | - |
| Other | 7 (3.0) | 10 (4.3) | 6 (2.6) | 8 (3.4) | 9 (3.8) | 0.50 | .335 |
| Low | 54 (23.0) | 54 (23.0) | 53 (22.6) | 50 (21.3) | 48 (20.4) | 0.92 | .815 |
| High | 78 (33.2) | 83 (35.3) | 85 (36.2) | 79 (33.6) | 68 (28.9) | 0.82 | .550 |
| H1 |  |  |  |  |  |  |  |
| No use | 96 (40.9) | 88 (37.5) | 91 (38.7) | 98 (41.7) | 110 (46.8) | - | - |
| Other | 7 (3.0) | 10 (4.3) | 6 (2.6) | 8 (3.4) | 9 (3.8) | 0.51 | .344 |
| Low | 79 (33.6) | 81 (34.5) | 83 (35.3) | 85 (36.2) | 76 (32.3) | 0.82 | .512 |
| High | 53 (22.6) | 56 (23.8) | 55 (23.4) | 44 (18.7) | 40 (17.0) | 0.98 | .963 |
| M1 |  |  |  |  |  |  |  |
| No use | 96 (40.9) | 88 (37.5) | 91 (38.7) | 98 (41.7) | 110 (46.8) | - | - |
| Other | 7 (3.0) | 10 (4.3) | 6 (2.6) | 8 (3.4) | 9 (3.8) | 0.51 | .339 |
| Low | 6 (2.6) | 7 (3.0) | 8 (3.4) | 7 (3.0) | 6 (2.6) | 0.57 | .441 |
| High | 126 (53.6) | 130 (55.3) | 130 (55.3) | 122 (51.9) | 110 (46.8) | 0.88 | .663 |
| A1 |  |  |  |  |  |  |  |
| No use | 96 (40.9) | 88 (37.5) | 91 (38.7) | 98 (41.7) | 110 (46.8) | - | - |
| Other | 7 (3.0) | 10 (4.3) | 6 (2.6) | 8 (3.4) | 9 (3.8) | 0.50 | .337 |
| Low | 68 (28.9) | 68 (28.9) | 68 (28.9) | 70 (29.8) | 60 (25.5) | 0.92 | .790 |
| High | 64 (27.2) | 69 (29.4) | 70 (29.8) | 59 (25.1) | 56 (23.8) | 0.81 | .534 |
| A2 |  |  |  |  |  |  |  |
| No use | 96 (40.9) | 88 (37.5) | 91 (38.7) | 98 (41.7) | 110 (46.8) | - | - |
| Other | 7 (3.0) | 10 (4.3) | 6 (2.6) | 8 (3.4) | 9 (3.8) | 0.51 | .342 |
| Low | 84 (35.7) | 83 (35.3) | 85 (36.2) | 77 (32.8) | 68 (28.9) | 0.98 | .950 |
| High | 48 (20.4) | 54 (23.0) | 53 (22.6) | 52 (22.1) | 48 (20.4) | 0.72 | .351 |

Receptor abbreviations as shown in S1 Table.

^a^Estimated using multivariate conditional logistic regression. Adjusted for physical illnesses and concomitant medications that remained in the final model in Table 2 (Cardiovascular disease except hypertension, diabetes mellitus, chronic hepatic disease, use of beta-blocking agents, calcium channel blockers, drugs used in diabetes, nasal preparations, antihistamines for systemic use and benzodiazepines use).

S1 Table C. Risk of other stroke with antipsychotic use within 14-day risk period by the receptor-binding profiles of the antipsychotic drugs (n = 121)

| Receptor-binding profile | Case  period  *N* (%) | Control period 1  *N* (%) | Control period 2  *N* (%) | Control  period 3  *N* (%) | Control period 4  *N* (%) | Adjusted risk ratio^a^ | *P* value^a^ |
| --- | --- | --- | --- | --- | --- | --- | --- |
| 5HT1A |  |  |  |  |  |  |  |
| No use | 29 (24.0) | 30 (24.8) | 29 (24.0) | 37 (30.6) | 35 (28.9) | - | - |
| Other | 6 (5.0) | 6 (5.0) | 5 (4.1) | 5 (4.1) | 8 (6.6) | 0.81 | .820 |
| Low | 40 (33.1) | 38 (31.4) | 41 (33.9) | 35 (28.9) | 40 (33.1) | 0.81 | .679 |
| High | 46 (38.0) | 47 (38.8) | 46 (38.0) | 44 (36.4) | 38 (31.4) | 0.89 | .802 |
| 5HT2A |  |  |  |  |  |  |  |
| No use | 29 (24.0) | 30 (24.8) | 29 (24.0) | 37 (30.6) | 35 (28.9) | - | - |
| Other | 6 (5.0) | 6 (5.0) | 5 (4.1) | 5 (4.1) | 8 (6.6) | 0.78 | .783 |
| Low | 49 (40.5) | 46 (38.0) | 49 (40.5) | 47 (38.8) | 49 (40.5) | 0.76 | .577 |
| High | 37 (30.6) | 39 (32.2) | 38 (31.4) | 32 (26.5) | 29 (24.0) | 0.97 | .957 |
| 5HT6 |  |  |  |  |  |  |  |
| No use | 29 (24.0) | 30 (24.8) | 29 (24.0) | 37 (30.6) | 35 (28.9) | - | - |
| Other | 6 (5.0) | 6 (5.0) | 5 (4.1) | 5 (4.1) | 8 (6.6) | 0.81 | .819 |
| Low | 70 (57.9) | 70 (57.9) | 70 (57.9) | 63 (52.1) | 65 (53.7) | 0.84 | .712 |
| High | 16 (13.2) | 15 (12.4) | 17 (14.1) | 16 (13.2) | 13 (10.7) | 0.92 | .899 |
| 5HT7 |  |  |  |  |  |  |  |
| No use | 29 (24.0) | 30 (24.8) | 29 (24.0) | 37 (30.6) | 35 (28.9) | - | - |
| Other | 6 (5.0) | 6 (5.0) | 5 (4.1) | 5 (4.1) | 8 (6.6) | 0.82 | .830 |
| Low | 54 (44.6) | 49 (40.5) | 53 (43.8) | 51 (42.2) | 50 (41.3) | 0.87 | .766 |
| High | 32 (26.5) | 36 (29.8) | 34 (28.1) | 28 (23.1) | 28 (23.1) | 0.84 | .732 |
| D2 |  |  |  |  |  |  |  |
| No use | 29 (24.0) | 30 (24.8) | 29 (24.0) | 37 (30.6) | 35 (28.9) | - | - |
| Other | 6 (5.0) | 6 (5.0) | 5 (4.1) | 5 (4.1) | 8 (6.6) | 0.82 | .824 |
| Low | 36 (29.8) | 35 (28.9) | 35 (28.9) | 35 (28.9) | 32 (26.5) | 0.84 | .728 |
| High | 50 (41.3) | 50 (41.3) | 52 (43.0) | 44 (36.4) | 46 (38.0) | 0.87 | .765 |
| D4 |  |  |  |  |  |  |  |
| No use | 29 (24.0) | 30 (24.8) | 29 (24.0) | 37 (30.6) | 35 (28.9) | - | - |
| Other | 6 (5.0) | 6 (5.0) | 5 (4.1) | 5 (4.1) | 8 (6.6) | 0.81 | .820 |
| Low | 26 (21.5) | 28 (23.1) | 28 (23.1) | 29 (24.0) | 26 (21.5) | 0.66 | .438 |
| High | 60 (49.6) | 57 (47.1) | 59 (48.8) | 50 (41.3) | 52 (43.0) | 0.94 | .988 |
| H1 |  |  |  |  |  |  |  |
| No use | 29 (24.0) | 30 (24.8) | 29 (24.0) | 37 (30.6) | 35 (28.9) | - | - |
| Other | 6 (5.0) | 6 (5.0) | 5 (4.1) | 5 (4.1) | 8 (6.6) | 0.82 | .824 |
| Low | 53 (43.8) | 58 (47.9) | 55 (45.5) | 51 (42.2) | 50 (41.3) | 0.78 | .586 |
| High | 33 (27.3) | 27 (22.3) | 32 (26.5) | 28 (23.1) | 28 (23.1) | 1.11 | .849 |
| M1 |  |  |  |  |  |  |  |
| No use | 29 (24.0) | 30 (24.8) | 29 (24.0) | 37 (30.6) | 35 (28.9) | - | - |
| Other | 6 (5.0) | 6 (5.0) | 5 (4.1) | 5 (4.1) | 8 (6.6) | 0.80 | .809 |
| Low | 5 (4.1) | 4 (3.3) | 7 (5.8) | 6 (5.0) | 6 (5.0) | 0.42 | .481 |
| High | 81 (66.9) | 81 (66.9) | 80 (66.1) | 73 (60.3) | 72 (59.5) | 0.87 | .753 |
| A1 |  |  |  |  |  |  |  |
| No use | 29 (24.0) | 30 (24.8) | 29 (24.0) | 37 (30.6) | 35 (28.9) | - | - |
| Other | 6 (5.0) | 6 (5.0) | 5 (4.1) | 5 (4.1) | 8 (6.6) | 0.79 | .800 |
| Low | 36 (29.8) | 43 (35.5) | 43 (35.5) | 44 (36.4) | 44 (36.4) | 0.47 | .145 |
| High | 50 (41.3) | 42 (34.7) | 44 (36.4) | 35 (28.9) | 34 (28.1) | 1.37 | .529 |
| A2 |  |  |  |  |  |  |  |
| No use | 29 (24.0) | 30 (24.8) | 29 (24.0) | 37 (30.6) | 35 (28.9) | - | - |
| Other | 6 (5.0) | 6 (5.0) | 5 (4.1) | 5 (4.1) | 8 (6.6) | 0.76 | .764 |
| Low | 53 (43.8) | 51 (42.2) | 55 (45.5) | 51 (42.2) | 55 (45.5) | 0.72 | .503 |
| High | 33 (27.3) | 34 (28.1) | 32 (26.5) | 28 (23.1) | 23 (19.0) | 1.07 | .896 |

Receptor abbreviations as shown in S1 Table.

^a^Estimated using multivariate conditional logistic regression. Adjusted for physical illnesses and concomitant medications that remained in the final model in Table 2 (Cardiovascular disease except hypertension, diabetes mellitus, chronic hepatic disease, use of beta-blocking agents, calcium channel blockers, drugs used in diabetes, nasal preparations, antihistamines for systemic use and benzodiazepines use).

S1 Table D. Risk of ischemic stroke with antipsychotic use within 7-Day risk period by the receptor-binding profiles of the antipsychotic drugs (n = 446)

| Receptor-binding profile | Case  period  *N* (%) | Control period 1  *N* (%) | Control period 2  *N* (%) | Control  period 3  *N* (%) | Control period 4  *N* (%) | Adjusted risk ratio^a^ | *P* value^a^ |
| --- | --- | --- | --- | --- | --- | --- | --- |
| 5HT1A |  |  |  |  |  |  |  |
| No use | 143 (32.1) | 162 (36.3) | 171 (38.3) | 180 (40.4) | 165 (37.0) | - | - |
| Other | 10 (2.2) | 12 (2.7) | 11 (2.5) | 14 (3.1) | 14 (3.1) | 0.81 | .697 |
| Low | 126 (28.3) | 124 (27.8) | 118 (26.5) | 116 (26.0) | 122 (27.4) | 1.26 | .351 |
| High | 167 (37.4) | 148 (33.2) | 146 (32.7) | 136 (30.5) | 145 (32.5) | 1.61* | .045 |
| 5HT2A |  |  |  |  |  |  |  |
| No use | 143 (32.1) | 162 (36.3) | 171 (38.3) | 180 (40.4) | 165 (37.0) | - | - |
| Other | 10 (2.2) | 12 (2.7) | 11 (2.5) | 14 (3.1) | 14 (3.1) | 0.77 | .636 |
| Low | 171 (38.3) | 163 (36.6) | 153 (34.3) | 148 (33.2) | 146 (32.7) | 1.46 | .104 |
| High | 122 (27.4) | 109 (24.4) | 111 (24.9) | 104 (23.3) | 121 (27.1) | 1.41 | .159 |
| 5HT6 |  |  |  |  |  |  |  |
| No use | 143 (32.1) | 162 (36.3) | 171 (38.3) | 180 (40.4) | 165 (37.0) | - | - |
| Other | 10 (2.2) | 12 (2.7) | 11 (2.5) | 14 (3.1) | 14 (3.1) | 0.74 | .587 |
| Low | 227 (50.9) | 207 (46.4) | 199 (44.6) | 188 (42.2) | 198 (44.4) | 1.51 | .062 |
| High | 66 (14.8) | 65 (14.6) | 65 (14.6) | 64 (14.4) | 69 (15.5) | 1.16 | .620 |
| 5HT7 |  |  |  |  |  |  |  |
| No use | 143 (32.1) | 162 (36.3) | 171 (38.3) | 180 (40.4) | 165 (37.0) | - | - |
| Other | 10 (2.2) | 12 (2.7) | 11 (2.5) | 14 (3.1) | 14 (3.1) | 0.78 | .645 |
| Low | 194 (43.5) | 182 (40.8) | 172 (38.6) | 172 (38.6) | 174 (39.0) | 1.41 | .134 |
| High | 99 (22.2) | 90 (20.2) | 92 (20.6) | 80 (17.9) | 93 (20.9) | 1.51 | .115 |
| D2 |  |  |  |  |  |  |  |
| No use | 143 (32.1) | 162 (36.3) | 171 (38.3) | 180 (40.4) | 165 (37.0) | - | - |
| Other | 10 (2.2) | 12 (2.7) | 11 (2.5) | 14 (3.1) | 14 (3.1) | 0.78 | .651 |
| Low | 176 (39.5) | 161 (36.1) | 148 (33.2) | 154 (34.5) | 150 (33.6) | 1.65* | .035 |
| High | 117 (26.2) | 111 (24.9) | 116 (26.0) | 98 (22.0) | 117 (26.2) | 1.23 | .403 |
| D4 |  |  |  |  |  |  |  |
| No use | 143 (32.1) | 162 (36.3) | 171 (38.3) | 180 (40.4) | 165 (37.0) | - | - |
| Other | 10 (2.2) | 12 (2.7) | 11 (2.5) | 14 (3.1) | 14 (3.1) | 0.79 | .660 |
| Low | 90 (20.2) | 93 (20.9) | 81 (18.2) | 88 (19.7) | 88 (19.7) | 1.31 | .307 |
| High | 203 (45.5) | 179 (40.1) | 183 (41.0) | 164 (36.8) | 179 (40.1) | 1.50 | .073 |
| H1 |  |  |  |  |  |  |  |
| No use | 143 (32.1) | 162 (36.3) | 171 (38.3) | 180 (40.4) | 165 (37.0) | - | - |
| Other | 10 (2.2) | 12 (2.7) | 11 (2.5) | 14 (3.1) | 14 (3.1) | 0.85 | .766 |
| Low | 140 (31.4) | 138 (30.9) | 136 (30.5) | 126 (28.3) | 145 (32.5) | 1.22 | .386 |
| High | 153 (34.3) | 134 (30.0) | 128 (28.7) | 126 (28.3) | 122 (27.4) | 1.87* | .015 |
| M1 |  |  |  |  |  |  |  |
| No use | 143 (32.1) | 162 (36.3) | 171 (38.3) | 180 (40.4) | 165 (37.0) | - | - |
| Other | 10 (2.2) | 12 (2.7) | 11 (2.5) | 14 (3.1) | 14 (3.1) | 0.77 | .629 |
| Low | 13 (2.9) | 12 (2.7) | 11 (2.5) | 9 (2.0) | 11 (2.5) | 1.87 | .211 |
| High | 280 (62.8) | 260 (58.3) | 253 (56.7) | 243 (54.5) | 256 (57.4) | 1.42 | .100 |
| A1 |  |  |  |  |  |  |  |
| No use | 143 (32.1) | 162 (36.3) | 171 (38.3) | 180 (40.4) | 165 (37.0) | - | - |
| Other | 10 (2.2) | 12 (2.7) | 11 (2.5) | 14 (3.1) | 14 (3.1) | 0.85 | .766 |
| Low | 125 (28.0) | 133 (29.8) | 116 (26.0) | 119 (26.7) | 129 (28.9) | 1.21 | .417 |
| High | 168 (37.7) | 139 (31.2) | 148 (33.2) | 133 (29.8) | 138 (30.9) | 1.72* | .024 |
| A2 |  |  |  |  |  |  |  |
| No use | 143 (32.1) | 162 (36.3) | 171 (38.3) | 180 (40.4) | 165 (37.0) | - | - |
| Other | 10 (2.2) | 12 (2.7) | 11 (2.5) | 14 (3.1) | 14 (3.1) | 0.79 | .671 |
| Low | 170 (38.1) | 168 (37.7) | 160 (35.9) | 154 (34.5) | 161 (36.1) | 1.24 | .361 |
| High | 123 (27.6) | 104 (23.3) | 104 (23.3) | 98 (22.0) | 106 (23.8) | 1.79* | .021 |

Receptor abbreviations as shown in S1 Table.

^a^Estimated using multivariate conditional logistic regression. Adjusted for physical illnesses and concomitant medications that remained in the final model in Table 2 (Cardiovascular disease except hypertension, diabetes mellitus, chronic hepatic disease, use of beta-blocking agents, calcium channel blockers, drugs used in diabetes, nasal preparations, antihistamines for systemic use and benzodiazepines use).

S1 Table E. Risk of ischemic stroke with antipsychotic use within 28-day risk period by the receptor-binding profiles of antipsychotic drugs (n = 446)

| Receptor-binding profile | Case  period  *N* (%) | Control period 1  *N* (%) | Control period 2  *N* (%) | Control  period 3  *N* (%) | Control period 4  *N* (%) | Adjusted risk ratio^a^ | *P* value^a^ |
| --- | --- | --- | --- | --- | --- | --- | --- |
| 5HT1A |  |  |  |  |  |  |  |
| No use | 110 (24.7) | 127 (28.5) | 134 (30.0) | 148 (33.2) | 143 (32.1) | - | - |
| Other | 8 (1.8) | 12 (2.7) | 12 (2.7) | 12 (2.7) | 11 (2.5) | 0.53 | .337 |
| Low | 150 (33.6) | 143 (32.1) | 147 (33.0) | 133 (29.8) | 135 (30.3) | 1.19 | .486 |
| High | 178 (39.9) | 164 (36.8) | 153 (34.3) | 153 (34.3) | 157 (35.2) | 1.36 | .227 |
| 5HT2A |  |  |  |  |  |  |  |
| No use | 110 (24.7) | 127 (28.5) | 134 (30.0) | 148 (33.2) | 143 (32.1) | - | - |
| Other | 8 (1.8) | 12 (2.7) | 12 (2.7) | 12 (2.7) | 11 (2.5) | 0.50 | .303 |
| Low | 202 (45.3) | 187 (41.9) | 178 (39.9) | 163 (36.6) | 167 (37.4) | 1.35 | .204 |
| High | 126 (28.3) | 120 (26.9) | 122 (27.4) | 123 (27.6) | 125 (28.0) | 1.13 | .644 |
| 5HT6 |  |  |  |  |  |  |  |
| No use | 110 (24.7) | 127 (28.5) | 134 (30.0) | 148 (33.2) | 143 (32.1) | - | - |
| Other | 8 (1.8) | 12 (2.7) | 12 (2.7) | 12 (2.7) | 11 (2.5) | 0.46 | .249 |
| Low | 264 (59.2) | 240 (53.8) | 227 (50.9) | 215 (48.2) | 224 (50.2) | 1.35 | .186 |
| High | 64 (14.4) | 67 (15.0) | 73 (16.4) | 71 (15.9) | 68 (15.3) | 0.87 | .658 |
| 5HT7 |  |  |  |  |  |  |  |
| No use | 110 (24.7) | 127 (28.5) | 134 (30.0) | 148 (33.2) | 143 (32.1) | - | - |
| Other | 8 (1.8) | 12 (2.7) | 12 (2.7) | 12 (2.7) | 11 (2.5) | 0.50 | .307 |
| Low | 228 (51.1) | 211 (47.3) | 205 (46.0) | 190 (42.6) | 190 (42.6) | 1.32 | .238 |
| High | 100 (22.4) | 96 (21.5) | 95 (21.3) | 96 (21.5) | 102 (22.9) | 1.13 | .670 |
| D2 |  |  |  |  |  |  |  |
| No use | 110 (24.7) | 127 (28.5) | 134 (30.0) | 148 (33.2) | 143 (32.1) | - | - |
| Other | 8 (1.8) | 12 (2.7) | 12 (2.7) | 12 (2.7) | 11 (2.5) | 0.52 | .325 |
| Low | 199 (44.6) | 186 (41.7) | 174 (39.0) | 172 (38.6) | 167 (37.4) | 1.42 | .148 |
| High | 129 (28.9) | 121 (27.1) | 126 (28.3) | 114 (25.6) | 125 (28.0) | 1.07 | .804 |
| D4 |  |  |  |  |  |  |  |
| No use | 110 (24.7) | 127 (28.5) | 134 (30.0) | 148 (33.2) | 143 (32.1) | - | - |
| Other | 8 (1.8) | 12 (2.7) | 12 (2.7) | 12 (2.7) | 11 (2.5) | 0.52 | .321 |
| Low | 96 (21.5) | 99 (22.2) | 93 (20.9) | 93 (20.9) | 93 (20.9) | 1.13 | .650 |
| High | 232 (52.0) | 208 (46.6) | 207 (46.4) | 193 (43.3) | 199 (44.6) | 1.33 | .231 |
| H1 |  |  |  |  |  |  |  |
| No use | 110 (24.7) | 127 (28.5) | 134 (30.0) | 148 (33.2) | 143 (32.1) | - | - |
| Other | 8 (1.8) | 12 (2.7) | 12 (2.7) | 12 (2.7) | 11 (2.5) | 0.56 | .390 |
| Low | 158 (35.4) | 150 (33.6) | 150 (33.6) | 146 (32.7) | 159 (35.7) | 1.07 | .790 |
| High | 170 (38.1) | 157 (35.2) | 150 (33.6) | 140 (31.4) | 133 (29.8) | 1.62 | .065 |
| M1 |  |  |  |  |  |  |  |
| No use | 110 (24.7) | 127 (28.5) | 134 (30.0) | 148 (33.2) | 143 (32.1) | - | - |
| Other | 8 (1.8) | 12 (2.7) | 12 (2.7) | 12 (2.7) | 11 (2.5) | 0.50 | .305 |
| Low | 14 (3.1) | 12 (2.7) | 10 (2.2) | 10 (2.2) | 11 (2.5) | 2.18 | .149 |
| High | 314 (70.4) | 295 (66.1) | 290 (65.0) | 276 (61.9) | 281 (63.0) | 1.24 | .342 |
| A1 |  |  |  |  |  |  |  |
| No use | 110 (24.7) | 127 (28.5) | 134 (30.0) | 148 (33.2) | 143 (32.1) | - | - |
| Other | 8 (1.8) | 12 (2.7) | 12 (2.7) | 12 (2.7) | 11 (2.5) | 0.55 | .373 |
| Low | 136 (30.5) | 138 (30.9) | 135 (30.3) | 130 (29.2) | 134 (30.0) | 1.08 | .769 |
| High | 192 (43.1) | 169 (37.9) | 165 (37.0) | 156 (35.0) | 158 (35.4) | 1.49 | .110 |
| A2 |  |  |  |  |  |  |  |
| No use | 110 (24.7) | 127 (28.5) | 134 (30.0) | 148 (33.2) | 143 (32.1) | - | - |
| Other | 8 (1.8) | 12 (2.7) | 12 (2.7) | 12 (2.7) | 11 (2.5) | 0.52 | .330 |
| Low | 201 (45.1) | 190 (42.6) | 191 (42.8) | 173 (38.8) | 178 (39.9) | 1.19 | .471 |
| High | 127 (28.5) | 117 (26.2) | 109 (24.4) | 113 (25.3) | 114 (25.6) | 1.43 | .183 |

Receptor abbreviations as shown in S1 Table.

^a^Estimated using multivariate conditional logistic regression. Adjusted for physical illnesses and concomitant medications that remained in the final model in Table 2 (Cardiovascular disease except hypertension, diabetes mellitus, chronic hepatic disease, use of beta-blocking agents, calcium channel blockers, drugs used in diabetes, nasal preparations, antihistamines for systemic use and benzodiazepine use).

Appendix. List of first- and second-generation antipsychotics included in this study: drug (ATC code):

1. First-generation (FGAs) (19 drugs): **Chlorpromazine (N05AA01)**, levomepromazine (N05AA02), fluphenazine (N05AB02), perphenazine (N05AB03), trifluoperazine (N05AB06), thioridazine (N05AC02), pipotiazine (N05AC04), **haloperidol (N05AD01)**, moperone (N05AD04), **flupentixol (N05AF01)**, clopenthixol (N05AF02), chlorprothixene (N05AF03), tiotixene (N05AF04), zuclopenthixol (N05AF05), pimozide (N05AG02), penfluridol (N05AG03), loxapine (N05AH01), **sulpiride (N05AL01)**, clotiapine (N05AH06; N05AX09)
2. Second-generation (SGAs) (9 drugs): Ziprasidone (N05AE04), **clozapine (N05AH02)**, **olanzapine (N05AH03)**, **quetiapine (N05AH04)**, **amisulpride (N05AL05)**, **risperidone (N05AX08)**, **zotepine (N05AX11)**, **aripiprazole (N05AX12)**, paliperidone (N05AX13)

The drugs in **bold** were substantially used among patients with schizophrenia in Taiwan and were also available for receptor-affinity analysis.
